# 肺癌微波消融治疗进展

**DOI:** 10.3779/j.issn.1009-3419.2010.01.15

**Published:** 2010-01-20

**Authors:** 强 王

**Affiliations:** 271000 泰安，中国人民解放军88医院肿瘤中心 Department of Oncology, PLA 88th Hospital, Tai'an 271000, China

肺癌(lung carcinoma)是目前全球死亡率最高的恶性肿瘤，在我国已成为第一大癌症，是我国肿瘤致死的第一大病因。美国每年大约有170 000人死于肺癌，占整个癌症死亡总数的30%^[1]^。外科手术肿瘤切除被公认为最为有效的方法。但大多数的肺癌患者就诊时已是晚期，肿瘤常多发或贴近血管，加之患者多为老年人，心肺功能差，临床上仅有15%的患者适合手术切除达到根治性治疗^[2]^。化学治疗、放射治疗和外科治疗手段不断发展，基因治疗、分子靶向治疗、免疫治疗也不断进步，但肺癌患者总的生存率仍然没有明显的改善，5年生存率不到15%。近年来，影像引导下的肿瘤微创消融治疗在国内外发展迅速，成为临床肿瘤治疗的重要手段^[3]^。目前，常用的微创热疗手段包括射频、微波、冷冻消融^[4]^，本文对近年来肺癌的微波消融研究进展综述如下。

## 1 微波对肿瘤治疗原理

微波是一种频率为300 MHz-300 GHz、波长为1 mm-1 m范围的高频电磁波，临床上最常用的微波频率为2 450 MHz，波长为12.25 cm。微波波束可被适当形态的天线聚焦，使能量集中，这一特性使之用于进行局部加温治疗，适于肿瘤局部微创治疗。治疗原理：①生物物理效应：微波作为生物物理效应能量，使极性分子随微波频率旋转摆动，同时其中的离子及所带胶状粒随微波运动而产生摩擦和热，通过热凝团使细胞核和染色质凝固，蛋白质凝固及细胞染色体畸变而诱导细胞死亡^[5]^。从光镜下可见大片癌细胞变性、坏死、核固缩、核仁消失。高温使癌细胞浆内溶酶体的活性增高，并产生新的溶酶体而使其自溶；②调节免疫作用：微波热凝固尚可产生Th-1细胞(T细胞和/或NK细胞)依赖的抗肿瘤免疫，推论肿瘤细胞经微波凝固后释放出抗原，或产生正常组织抗体，即所谓自身抗体，导致自身免疫反应，减弱了肿瘤的远隔侵袭作用并破坏了肿瘤细胞分泌的封闭抑制因子对免疫系统抑制，增强抗肿瘤免疫，降低了肿瘤的转移率。热疗还可使热休克蛋白HSP70的表达上调，从而提高抗肿瘤免疫作用，诱导肿瘤细胞死亡^[6]^。微波热效应可增加局部血液和淋巴循环，受照射组织代谢加强，使细胞内cAMP增加，改善营养，从而加速了组织的再生和修复能力，还可以提高组织的免疫反应能力；③解剖因素：一般认为肿瘤组织的微血管呈不规则分布，阻力大，散热慢，且肿瘤细胞较正常细胞含水量高，可吸入较多的微波能量，加温后瘤组织升温较快，肿瘤细胞在高温下可发生血流停滞，淤血缺氧，细胞色素氧化酶活性降低，内呼吸抑制；正常组织微循环健全，散热较快。在瘤体温度升至45 ℃以上时，周围正常组织的温度仍在40 ℃以下；④基因水平调节：微波还能抑制癌细胞的DNA、RNA和蛋白质合成^[7]^，激活溶酶体酶，紊乱细胞骨架排列，使细胞膜的通透性增高，膜内低分子蛋白外溢，导致癌细胞破坏。因此，高温对肿瘤细胞具有选择性杀灭作用，对正常细胞则杀伤作用较小。

## 2 肺癌微波消融治疗的实验研究

胡海洋等^[8]^采用组织块悬液注射法在30只新西兰大白兔中建立实验性VX2肺癌模型，随机分为对照组、治疗A组(40 W×120 s)、B组(80 W×60 s)。在CT引导下经皮穿刺，并按预设参数进行微波热凝治疗，对照组予以假性治疗。观察实验兔术后一般状况，定期CT检查观察肿瘤进展情况，在治疗后第0、1、7天每组随机解剖1只实验兔观察局部组织学改变。结果发现，肿瘤细胞种植成功率为100%。肺癌模型制备成功率为86.7%(26/30)。对照组、治疗A组和B组的生存期分别为(39.7±5.3)天、(62.2±4.4)天和(61.7±4.5)天(*P* < 0.01)。气胸的发生情况分别为33.3%(2/6)、55.6%(5/9)和33.3%(3/9)(*P* > 0.10)。证明微波消融治疗是一种安全、有效、微创的肺癌治疗方法。Furukawa等^[9]^选用小猎犬行微波消融肺组织实验，采用的微波频率为2 450 MHz，电极针直径约1.6 mm。结果显示，消融范围直径约2.0 cm，距瘤中心1.0 mm处温度可达70 ℃-80 ℃；观察6个月，无咳血、气胸等并发症。微波消融即刻电极周围组织病理学发生改变：胶原纤维变性、增厚，6个月后其消融肺组织被瘢痕纤维组织所取代。耿怀成、陈龙邦等^[10]^在CT引导下将微波天线插入活体猪肺的不同位置，采用不同的辐射功率/时间组合进行消融，并观察术后CT表现。结果证实60 W/5 min消融灶均值为31.2 mm×22.3 mm；60 W/10 min为35.4 mm×65.2 mm；80 W/5 min为40.2 mm×32.4 mm；80 W/10 min为45.2 mm×40.5 mm；光镜下见消融区出血性坏死，坏死区外围有水肿带、炎细胞侵润等改变，术后CT扫描及处死动物未见有气胸、血胸并发症。目前动物实验表明，采用较大功率的微波、治疗时间适当延长，有可能灭活直径在5 cm以下的肿瘤，但肿瘤组织的血供、含水量与正常肺组织有较大差异，必然导致微波消融范围的差别。曹冰生等^[11]^证明组织含水量大微波消融范围也增大，增加组织中的水分含量可降低微波消融天线的杆温，减少中心炭化，增大消融范围，消融形状更接近球形。动物实验中消融的活体猪肺组织内有大量的中性粒细胞、淋巴细胞，证明微波组织消融治疗后能刺激机体的免疫功能。

## 3 肺癌微波消融治疗的临床研究进展

目前，微波在肺癌中的应用主要有两种形式：经纤维支气管镜微波消融治疗、经皮穿刺微波消融治疗(percutaneous microwave coagulation therapy, PMCT)。

### 3.1 经纤维支气管镜微波消融治疗中心型肺癌

中心型肺癌占肺癌的70%，多阻塞大气道，导致阻塞性肺不张及难治性阻塞性肺炎，解除阻塞为姑息治疗的重要办法。适应症：中心型肺癌伴支气管阻塞而又不适于手术治疗者；肺癌术后复发伴有支气管阻塞者；气道内良性肿瘤或肉芽肿；各种原因所致的气道内狭窄；纤支镜可视范围内的出血。禁忌证：气管重度狭窄、气道外压狭窄、周围性病变、弥漫性出血、合并有出血性疾病等。邓毅书等^[12]^报道纤维支气管镜下微波消融治疗20例中心型肺癌，有效率[完全缓解(complete relief, CR)+部分缓解(partial relief, PR)+好转(minor relief, MR)]为80%，Karnofsky评分：治疗后增加了(12.5±5.72)分。*P* < 0.05，治疗后生存期 > 2年的7例。对管内型中心型非小细胞肺癌，微波消融是一种有效的局部治疗方法，能改善症状，提高生活质量。曹慧等^[13]^报道纤维支气管镜下微波消融治疗46例中心型肺癌所有病例治疗后瘤体缩小，症状明显改善，气管通畅，总有效率为95.6%，无严重并发症发生。经纤维支气管镜微波消融治疗中心型肺癌的机理是通过特制的袖状针探头将高能量密度的微波集中于尖端，其产生温度达60 ℃-120 ℃，直接作用于癌病灶，由于癌细胞对温度的敏感性较正常细胞高，通常在42 ℃-45 ℃被杀伤，60 ℃-100 ℃癌组织炭化，能有效缓解管内型中心型肺癌患者局部阻塞表现，减轻痛苦，提高生活质量，延长生存期。利用微波治疗肺癌是控制原发病灶较理想手段之一，其中经内镜微波治疗是一种姑息拓通气道的十分有效方法，表现为临床症状改善、咯血停止、精神好转、呼吸困难消失或减轻、肺不张复张。近期效果是显著的，尤其是对腔内肿瘤效果尤甚。且微波消融治疗边界清楚，无炭化，止血效果好，安全性极佳。

### 3.2 经皮穿刺微波消融治疗(PMCT)周围型肺癌

对于周围型肺癌，在CT引导下经皮穿刺微波消融治疗周围型肺癌对早期原发性肺癌(Ⅰ期)可达根治效果，对肺内转移性肿瘤亦有很好疗效。Wolf等^[14]^报道CT引导下经皮穿刺微波消融治疗50例82个转移性肺癌，平均随访10个月、肿瘤直径 > 3 cm的患者，26%微波消融部位有肿瘤残余，22%的患者有肿瘤复发，1年局部控制率为67%，第1次复发时间平均为16.2个月。*Kaplan-Meier*生存曲线：消融1年存活率为65%，2年为55%，3年为45%。肿瘤特异死亡：1年存活率为83%，2年为73%，3年为61%，与肿瘤大小及有无肿瘤残留无明显关系。空洞形成(治疗肿瘤43%有空洞)与降低肿瘤特异死亡有关。冯威健等^[15]^使用针式单极微波辐射天线在CT引导下经皮肺穿刺微波消融周围型肺癌，以2 450 MHz的微波、65 W辐射60 s，对20例患者(原发性肺癌8例、转移性肺癌12例)的28个病灶进行治疗。随访(3-24)个月，16例患者健在。全部肿块均缩小，13个病灶缩小50%以上，3例病灶消失，有效率为57.1%。CT表现为即刻消融灶呈约3.5 cm× 2.5 cm的软组织影，1周后消融区域内见气化灶，外周有高密度反应区，1个月后消融区进一步缩小，3个月后实变，1年后几乎消失，治疗后细胞学证实肿瘤组织坏死，无明显副作用及并发症。He等^[16]^用超声引导微波消融治疗12例病人的16个肿瘤，并进行(6-40)个月随访(平均20个月)，7人无严重并发症存活，5人死于消融后转移。12例病人肿瘤体积均有缩小，彩色多普勒血流成像显示肿瘤血流量减少，造影剂增强性计算机断层X线照相证明9个肿瘤无增强，7个肿瘤部分增强降低。Padda^[17]^报道肺癌微波消融治疗的并发症有肿瘤残留、形成易于感染的空洞。Wasser等^[18]^认为肺癌微波消融治疗的主要理论优点包括肿瘤内连续的高温、较大的消融体积、治疗时间的缩短和改良的热传导元件优于当前的热消融技术。

## 4 肺癌微波消融联合其它治疗的应用

Grieco等^[19]^将内镜微波治疗联合放射疗法治疗肺癌，认为联合治疗的疗效明显高于单纯一种治疗，*Kaplan-Meier*生存曲线：生存率和局部复发率作为主要终点值。中位随访时间为19.5个月，6个月总生存率达97.6%，1年生存率达86.8%，2年生存率达70.4%，3年生存率57.1%。 < 3 cm的肿瘤11.8%平均在(45.6±4.1)个月出现局部复发， > 3 cm的肿瘤33.3%平均在(34.0±7.8)个月出现复发(*P*=0.03)。李球兵等^[20]^联合局部放疗与单纯局部放疗相关免疫指标变化，在纤维支气管镜(纤支镜)引导下微波组织消融联合局部放疗，治疗晚期中央型肺癌58例，治疗后1、2周内外周血树突状细胞数量、T淋巴细胞亚群、淋巴细胞转化率明显提高，癌胚抗原、肿瘤相关生长因子明显下降；治疗组30例，1周内气道阻塞完全缓解18例，部分缓解12例，有效率95%[CR+PR+无变化(normal control, NC)]，明显高于对照组(*P* < 0 05)。纤维支气管镜介导微波热消融疗法加局部放疗治疗晚期中心型肺癌能快速解除气道阻塞，并有效改善患者机体细胞的免疫功能。认为微波治疗可有效改善患者机体细胞的免疫功能。均取得了显著的疗效，与单用其它疗法比较效果明显提高，差异显著。胡鸿涛等^[21]^用微波联合放疗治疗周围型肺癌，其有效率、1年、2年、3年生存率均高于单纯放疗者。石寒冰等^[22]^将微波联合化疗与单用化疗治疗非小细胞肺癌进行疗效比较，均认为联合疗法的客观疗效及症状缓解均优于单纯化疗(两者化疗方案不同)，可以提高患者近期生存率。经纤支镜介入微波并局部化疗能较快地使肿瘤缩小或消失，肺不张复张。

## 5 问题和展望

近年来，影像引导下肿瘤热消融已证实是一种有发展前途且安全有效的治疗手段。目前，国内外常用的肺癌热消融手段除了微波还有射频和激光等。微波、射频及激光治疗肺癌均可对病灶原位有效灭活，减轻肿瘤负荷，近期疗效确切，中、远期疗效待观察分析。肺癌微波、射频及激光消融常见并发症有气胸、咳血、胸腔积液，少见并发症有肺出血、支气管胸膜瘘、肺部感染。射频消融不宜用于靠近大血管的肿瘤^[23]^。治疗费用高于微波消融。激光消融特别适用于大小为2.0 cm-3.0 cm位于肺中上部的病灶。其消融形状比射频合理，肺出血并发症低于射频。经皮穿刺微波消融治疗周围型肺癌或经纤维支气管镜微波消融治疗中心型肺癌，其优点在于对病灶原位灭活的同时，能限制对正常含气肺组织的损伤，微波热消融还可以增加局部血流和淋巴循环，加快组织再生和修复能力，提高机体免疫反应。气胸、皮肤烫伤、肺出血等并发症发生率已明显降低，无治疗相关死亡病例。微波消融治疗肺癌的缺点是虽然取得良好的肿瘤局部消融效果，但因肿瘤不规则或肿瘤体积较大存在消融不完全的可能，这些存活肿瘤细胞将成为肿瘤复发和转移的隐患。微波联合放疗治疗肺癌可以消灭微波消融的肿瘤残留问题并减少肿瘤细胞的热耐受性，提高微波消融治疗效果^[24]^。微波消融治疗肺癌是一种新兴肺癌微创热消融治疗模式，属局部治疗手段，进一步研究其与放疗、化疗、生物治疗、基因治疗等全身治疗手段的协同或互补作用机制，其理论将为肺癌治疗提供科学依据，以肺癌微波消融为基础的联合治疗方法将有希望成为减轻肺癌患者肿瘤负荷、减少肿瘤细胞远处转移、提高肺癌治疗疗效、延长癌症患者生存期和提高生活质量有效方法。
